# The effect of squat training with different eccentric contraction tempos on lower limb muscle strength

**DOI:** 10.1371/journal.pone.0354725

**Published:** 2026-07-27

**Authors:** Mushuai Hao, Haonan Qi, Liang Zhao, Wei Han

**Affiliations:** 1 School of Competitive Sports, Shandong Sport University, Rizhao, China; 2 College of Physical Education, Hebei Normal College, Shijiazhuang, China; Portugal Football School, Portuguese Football Federation, PORTUGAL

## Abstract

**Objective:**

This study aims to investigate the impact of 8-week squat training with different eccentric contraction tempos on maximal squat strength, jump height, and sprint performance among university students majoring in sports training.

**Methods:**

Thirty participants were randomly assigned to either the 4/0/X/0 group, the 2/0/X/0, or the self-selected eccentric, isometric and inter-repetition tempo group (V/V/X/V), performing tempo-specific loaded squat training twice weekly for 8 weeks. Maximal squat strength, 30-meter sprint performance, countermovement jump (CMJ), and squat jump (SJ) heights were assessed before and after the intervention. A two-way repeated-measures ANOVA and effect size analysis were used to evaluate the training outcomes.

**Results:**

1) For maximal squat strength, no statistically significant inter-group difference was detected (P > 0.05). All three groups exhibited significant within-group improvements relative to baseline (P < 0.05 to P < 0.01). Descriptive effect size analysis showed the magnitude of improvement ranked as follows: 2/0/X/0 group (ES = 1.45)> V/V/X/V group (ES = 0.85)> 4/0/X/0 group (ES = 0.64). 2) For CMJ height, both the 2/0/X/0 and V/V/X/V groups showed significant improvement compared to pre-intervention (P < 0.05), while the 4/0/X/0 group exhibited a significant decrease (P < 0.01). ES magnitudes followed the order: 2/0/X/0 group (ES = 1.07) > V/V/X/V group (ES = 0.30) > 4/0/X/0 group (ES = −0.90). 3) SJ height results mirrored CMJ trends, with ES values ranking:2/0/X/0 group (ES = 1.09) > V/V/X/V group (ES = 0.34) > 4/0/X/0 group (ES = −0.83). 4) In 30-meter sprint performance, the 2/0/X/0 group demonstrated significant improvement (P < 0.05), the 4/0/X/0 group showed significant decline (P < 0.01), and the V/V/X/V group exhibited no significant change. Effect sizes indicated significant improvement in the 2/0/X/0 group (ES = −0.76), no meaningful change in the V/V/X/V group (ES = −0.10), and a significant decline in the 4/0/X/0 group (ES = 0.63). Notably, despite significant gains in maximal strength, the 4/0/X/0 group displayed concurrent decrements in stretch-shortening cycle-based explosive performances.

**Conclusion:**

Compared with the 4/0/X/0 and V/V/X/V tempos, the 2/0/X/0 tempo showed the most favorable improvement trend in lower-limb maximal strength, and induced significantly greater enhancements in explosive power performance.

## Introduction

Resistance training not only promotes adaptability within the nervous system but also induces significant morphological changes in muscle. The adaptive changes in the nervous system are primarily reflected in the enhanced recruitment capabilities of motor units, improved coordination of intermuscular neural discharge, and the reduction of inhibitory mechanisms, such as Golgi tendon organs [1]. Morphological changes in muscle include an increase in muscle fiber cross-sectional area and an enhancement in tendon stiffness [2,3].

To achieve improvement in specific physical qualities, it is essential to precisely control numerous training variables. Over the years, an abundance of research on resistance training variables has emerged. In 2002, the American College of Sports Medicine (ACSM) published a comprehensive review summarizing resistance training models for healthy adults, detailing common training variables such as load volume, intensity, exercise order, rest intervals between sets, muscle contraction type, and movement tempo [3]. As a key multi-joint movement for lower-body development, the back squat operates within the functional kinetic chain and aligns with modern joint-by-joint training paradigms, which emphasize the integrated mobility and stability of ankle, knee, hip, and core structures for optimal force transmission and injury prevention. Such a framework further supports the need for precise manipulation of movement tempo to enhance specific adaptations in strength and explosive performance [4]. However, many scientific studies on resistance training often overlook movement tempo, a critical variable [5]. Movement tempo refers to the speed at which each movement repetition is performed. In resistance training, tempo is often expressed in numbers representing the time taken by muscles during different phases of contraction. For instance, 4/0/1/0 indicates a 4-second eccentric contraction, 0 seconds of isometric contraction or transition time, 1 second of concentric contraction, and the final digit denotes the pause duration after the concentric phase before the next repetition. It is important to note that “V” represents a self-selected tempo, while “X” denotes the fastest possible contraction speed [5]. When movement tempo changes, it affects muscle contraction duration (Time Under Tension, TUT), which refers to the sum of the durations of each muscle contraction phase. The prolongation of TUT can lead to the accumulation of blood lactic acid, growth hormone, and testosterone [6]. On the other hand, the magnitude of load directly influences movement tempo—the greater the load, the slower the movement tempo [5]. However, studies have pointed out that regardless of the training load, prompting athletes to exert force as fast and forcefully as possible is the key to driving high-speed strength adaptation [7–9].

Research on movement tempo can be traced back to 1993. Warren et al. [10] compared weighted squat exercises at fast and slow tempos and found that the power output of the fast tempo group was higher than that of the slow tempo group, but the latter had stronger maximum isometric muscle strength. Since then, a large number of studies have investigated movement tempo in resistance training [11–18]. Nevertheless, there is still controversy regarding which movement tempo has the best effect on maximum strength and explosive power [19–22]. Among numerous studies on movement tempo, eccentric contraction tempo has attracted the attention of many practitioners. Eccentric training, as a method to improve strength performance, has always been a research hotspot. A common view is that eccentric contraction strength is 120% to 160% of concentric contraction, and eccentric contraction can more efficiently promote protein synthesis reactions, and positively regulate anabolic signaling pathways and gene expression [6,23]. The choice of eccentric contraction tempo has an important impact on muscle strength. Wilk et al. [5] pointed out that for muscle hypertrophy, slower eccentric contractions combined with fast concentric contractions seem to achieve better results, while for the improvement of muscle strength, it is still unclear whether there is a specific tempo that is more effective than other tempos. Handford et al. [6] believe that an eccentric contraction tempo of less than 2 seconds can improve subsequent concentric contraction performance, and there is no significant difference in the impact on muscle strength between eccentric contraction tempos of 2–6 seconds. Adrián et al.[24] argue that a moderate eccentric tempo combined with a fast concentric tempo (3–4/0/1/0) can effectively improve maximum strength. The divergence of results may be attributed to three aspects: First, significant disparities in participants’ training levels (e.g., resistance-trained individuals, amateur athletes, elite athletes); Second, utilization of non-conventional training methods (e.g., > 6s ultra-long eccentric tempo)and non-functional single-joint/machine-based exercises–while widely utilized, multi-joint movements better reflect sport-specific motor patterns; Furthermore, inadequate control over key variables, particularly failure to isolate eccentric and concentric contraction tempos. When multi-phase tempos are mixed, it becomes impossible to discern whether strength gains originate from eccentric tempo variation or concentric rhythm alteration [5].

Based on this, it is necessary to precisely control the tempo of a certain contraction phase to further clarify the impact of specific variables on different muscle strength performance modes. In view of this, the tempo control in this study will focus on eccentric contraction. At the same time, most sports require extremely high concentric output power, and explosive contraction intention is the core of driving high-speed sports performance. Therefore, the concentric contraction tempo of all groups is set to X tempo.

This study hypothesizes that a 4-second eccentric contraction tempo combined with an explosive concentric contraction tempo can bring better effects on lower limb maximum strength; a 2-second eccentric contraction tempo combined with an explosive concentric contraction tempo can bring better effects on explosive power indicators; the self-selected tempo group can also increase maximum strength, but the increase in explosive power is not significant.

## Methods

### Experimental approach to the problem

This study adopted a randomized controlled trial design, with participants randomly assigned to the 2/0/X/0 group, the 4/0/X/0 group, and the V/V/X/V group. The allocation and randomization were conducted by a researcher blinded to the participants’ baseline conditions. To determine the required sample size, an a priori power analysis was performed prior to the experiment using G*Power software (version 3.1.9.2, Düsseldorf, Germany). The analysis was specified as an F-test for repeated-measures ANOVA (within-between interaction), with input parameters set as follows: effect size *f* = 0.3, α = 0.05, statistical power = 0.8, 3 independent groups, and 2 repeated measurements. The calculation returned a required total sample size of 30 [25]. Accordingly, a total of 30 participants were finally included in this study.

The V/V/X/V group was defined as follows: eccentric tempo (V), isometric tempo (V), and inter‑repetition interval (V) were self‑selected by each participant, while the concentric phase was uniformly set to maximal explosive effort (X) in all three groups.

The study focuses on the effects of squat training with different eccentric contraction tempos on the lower limb muscle strength of university students majoring in sports training.

### Subjects

Thirty college students majoring in sports training (age: 19.9 ± 1.5 years; height: 180.3 ± 3.0 cm; weight: 76.1 ± 5.6 kg) volunteered to participate in this study from October 1, 2023, to December 31, 2023 (see Table 3 for subject information). All participants had more than 4 years of professional training experience (in track and field, boxing, rowing, football, etc.). Although they possessed considerable training backgrounds, they represented diverse sport specializations, with squat 1RM mainly ranging from 1.5 to 2.0 times body weight. The criterion of ≥ 1.5 times body weight was adopted to ensure adequate sample size and consistent baseline strength. This study was approved by the Ethics Committee of Shandong Sport University (approval number: 2022031) and conforms to the ethical standards for research involving human subjects as specified in the Declaration of Helsinki. Finally, the subjects were fully informed of the potential risks associated with the study, and all of them accepted and signed the informed consent form. The inclusion criteria for the subjects were as follows:

1. Having at least 2 years of strength training experience;
2. Maximum squat strength being no less than 1.5 times their own body weight;
3. Being in good health, with no lower limb injuries in the past 6 months and no other diseases;
4. Volunteering to participate;
5. Refraining from consuming stimulants such as caffeine and alcohol during the experiment.

### Procedures

**Fig 1:**
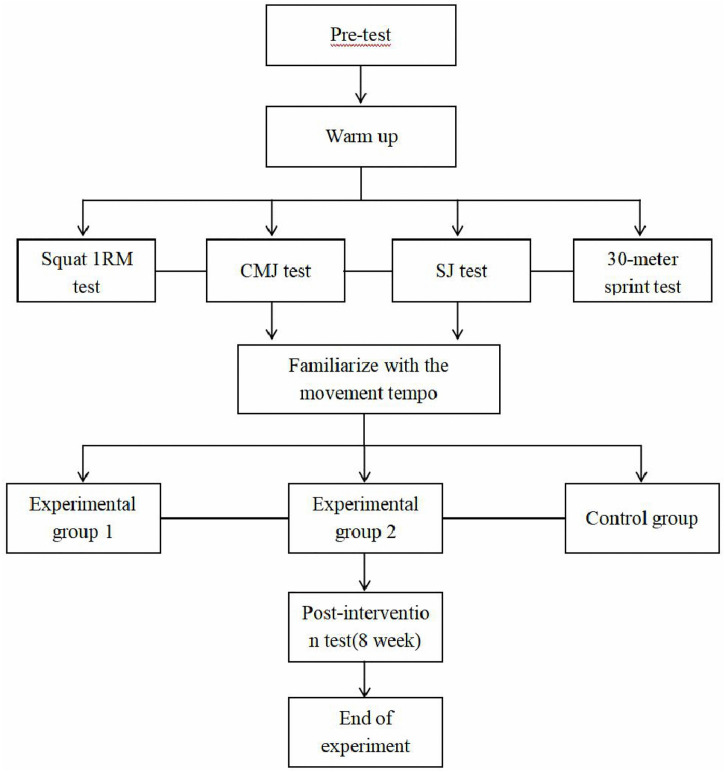
Experimental procedure flow chart.

To assess lower limb muscle strength, the one-repetition maximum (1RM) for the squat was measured. To evaluate lower limb explosive power, the countermovement jump (CMJ), squat jump (SJ), and 30-meter sprint were measured. The experiment lasted 10 weeks, with the first week serving as the pre-test phase, weeks 2–9 being the formal intervention, and the last week being the post-test. All indicators were tested in two sessions: the first session focused on the squat 1RM test. To minimize the potential impact of maximum strength testing on explosive power indicators, measurements of CMJ, SJ height, and 30-meter sprint were conducted 48 hours later. A 10-minute interval was maintained between the three tests to ensure nearly complete recovery of the ATP-CP system. All tests were administered by the same experienced strength and conditioning coach. One day after all indicator tests were completed, the participants were gathered to familiarize themselves with the rhythm of the squat movement through practice (The flowchart is shown in Fig 1).

### Squat 1 RM

The Maximum strength test follows the standard procedures of National Strength and Conditioning Association (NSCA), and the specific operations are as follows:

1. Instruct the participants to perform a warm-up with a light load that allows them to complete 5–10 repetitions with ease.
2. Rest for 1 minute.
3. Estimate a warm-up load that the participant can complete for 3–5 repetitions by increasing the load using the following increments: 14–18 kg for lower body exercises or 10%–20% of the estimated maximum load.
4. Rest for 2 minutes.
5. Estimate a submaximal load that the participant can complete for 2–3 repetitions, using the same incremental approach:14–18 kg for lower body exercises or 10%–20% of the estimated maximum load.
6. Rest for 2–4 minutes.
7. Continue to progressively increase the load in the following manner: 14–18 kg for lower body exercises or 10%–20% of the estimated maximum load.
8. Instruct the participant to attempt a 1RM (one repetition maximum).
  - If successful, rest for 2–4 minutes, then return to step 7.
  - If unsuccessful, rest for 2–4 minutes, then reduce the load using the following increments: 7–9 kg or 5%–10%
9. Return to step 8 and continue adjusting the load, increasing or decreasing as needed, until the participant successfully completes one repetition with proper technique. Ideally, the 1RM should be determined within 3–5 testing sets.

Previous research has supported the reliability of 1RM and 4–6RM assessment in both free-weight and machine-based squats, and has demonstrated significant associations between body mass and maximal strength performance in male athletes from diverse sport specialties [26].

### CMJ and SJ tests

**Fig 2:**
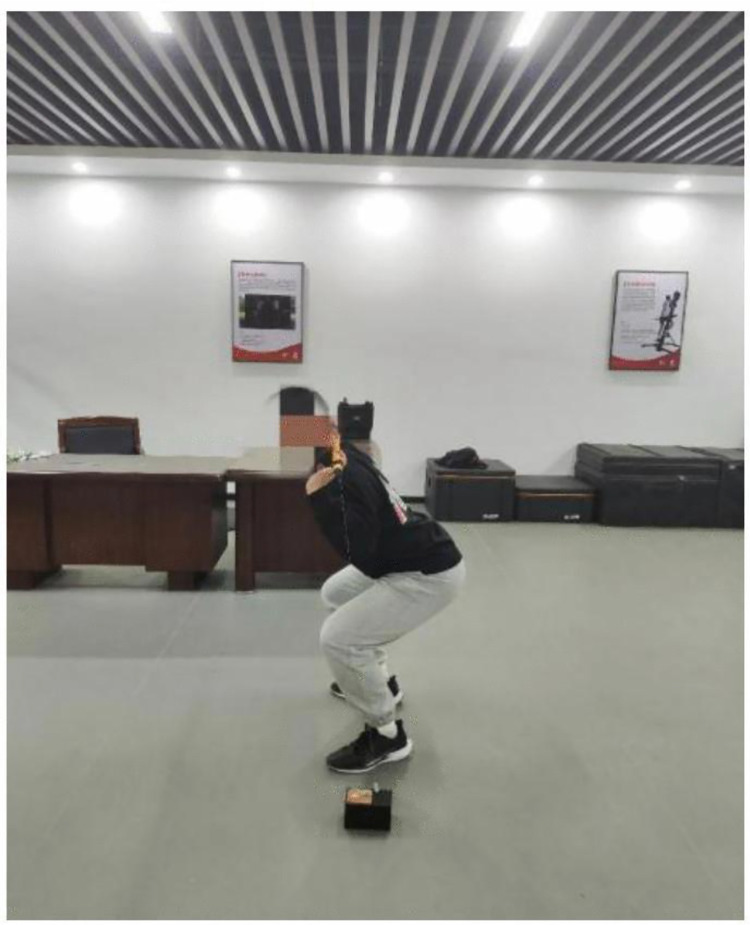
Vertical jump test.

**Fig 3:**
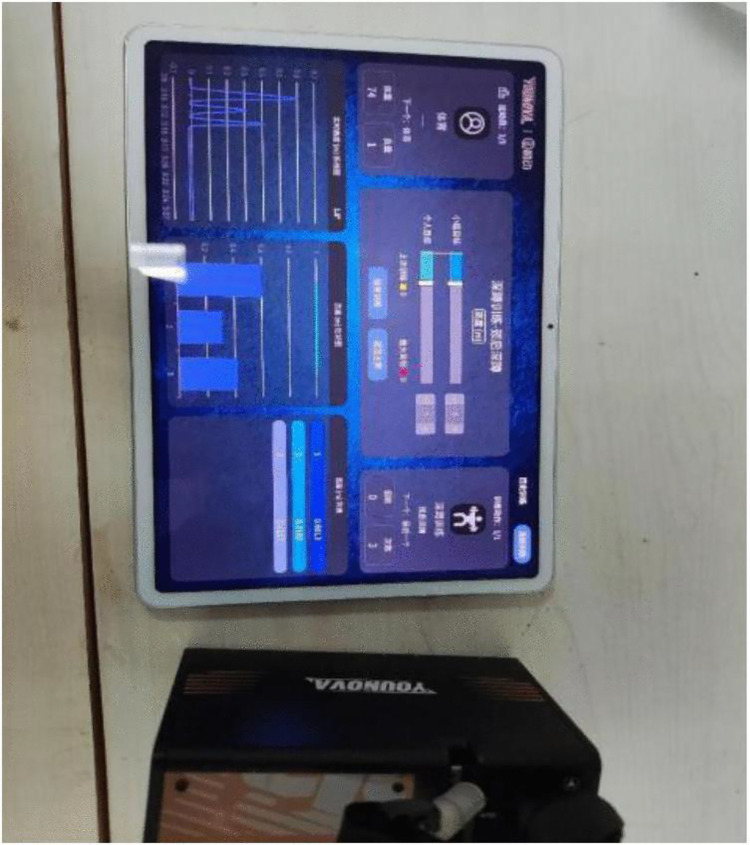
VBT supporting APP.

A linear position transducer (VBT Pro) was used to measure the heights of no-arm-swing CMJ and SJ variants, which has been validated as a reliable alternative to force plates [27,28]. The tablet app paired with the sensor was activated, with the test index set to height (cm) and the test movement set to vertical jump. The fixture of the sensor cable was attached to one end of a PVC pole, which subjects then placed across the upper trapezius muscles, holding the pole in place with relaxed hands while standing still. The app was used to zero the PVC pole’s position, and the jump height test commenced (see Figs 2 and 3) Subjects were instructed to avoid excessive forward or backward movement during the jump to ensure a vertical leap as high as possible. The entire process was supervised by two experienced physical fitness testers. Each movement was tested three times, with the highest value recorded, and a three-minute rest period was allowed between each movement and each jump to ensure adequate recovery. The PVC pole placed across the upper trapezius was a deliberate methodological choice: it eliminates the propulsive contribution of arm swing, thereby enabling a more isolated and objective reflection of lower-extremity explosive capacity.

During all jump tests, participants were explicitly instructed to jump as high as possible using lower-body push-off while avoiding trunk flexion, with strict and consistent supervision provided by experienced strength and conditioning coaches. However, individual differences in trunk and lower limb anthropometry may unavoidably influence trunk inclination during the downward movement, which represents a minor methodological consideration inherent to this measurement approach.

Notably, methodological complexities and potential sources of error exist in power and jump height assessment even when using well-established devices such as force plates, highlighting the importance of standardized testing protocols to minimize bias [29].

### 30-meter sprint

The 30-meter sprint was tested using a four-gate infrared speed sensitivity testing system (Brower Timing System, USA). Before the test, two researchers placed a pair of infrared timing gates at the 30-meter start and finish lines. After warming up, subjects stood ready at the starting line and sprinted 30 meters as fast as possible at the sound of a whistle. Subjects completed 3 sprints, with 3–5 minute rest intervals between attempts, and the best performance was recorded as the final data.

### Intervention

To maximize performance and minimize the risk of injury during the experiment, all subjects were required to perform a standardized warm-up before each session, consisting of general warm-up, dynamic stretching, and specific warm-up. The general warm-up involved 5 minutes of jogging to raise body temperature and reduce muscle viscosity. Dynamic stretching included bodyweight squats with thoracic spine rotation, lunging with twists, and lateral lunges. Since the experiment involved squat training, the specific warm-up consisted of light-load squats, conducted in three sets, with progressively increasing loads that the subjects could comfortably complete (see Table 1 for the specific warm-up protocol). During the experiment, two individuals with extensive experience in strength training supervised the standardization and movement tempo of the exercises. One monitor controlled the tempo using a stopwatch; if a participant failed to maintain the prescribed tempo, the set was terminated. The other supervisor ensured proper range of motion, strictly prohibiting any bouncing during the concentric contraction phase. Verbal encouragement was provided consistently throughout the protocol.

**Table 1:** Experimental warm-up protocol.

Specific Warm-Up Protocol
1. jogging for 5 minutes
2. Bodyweight sqats+thoracic spine rotation ×10 reps
3. Lunging with twists x 10 reps
4. Lateral lunges x 10 reps
5. Light-load squats 3 sets x 10 reps

Training load is a primary determinant of strength adaptation. The National Strength and Conditioning Association (NSCA) recommends loads ≥85% of 1RM for maximizing muscle strength [30].While debates persist regarding optimal load parameters for strength development [31], this discussion exceeds the scope of the present investigation. Given that participants had substantial athletic experience but no prior exposure to tempo-controlled resistance training, a conservative load of 70% of 1RM was selected to prioritize safety. Although NSCA guidelines recommend loads of ≥85%1RM for maximizing maximal strength, a load of 70%1RM was employed in this study for three methodological reasons. First, all participants were experienced in resistance training but had no prior experience with strict tempo-controlled squat training; a moderate load ensured consistent movement tempo and proper technique across all groups. Second, the study compared conditions with markedly different time under tension (TUT); a conservative load reduced excessive fatigue, minimized injury risk, and allowed stable implementation of the prescribed tempos. Third, progressive overload was strictly applied using the 2-for-2 rule, which guaranteed sufficient training stimulus to improve maximal strength despite the moderate absolute load. Progressive overload was implemented using the NSCA’s “2-for-2 rule”: if a participant completed two additional repetitions beyond the target in the final set of two consecutive sessions, the load was increased by 5%−10% [30]. This approach ensured systematic progression while minimizing injury risk. Following 8 weeks of intervention, the group-averaged final training loads (mean ± SD) were 105.50 ± 7.53 for the 2/0/X/0 group, 103.50 ± 9.14 for the 4/0/X/0 group. The detailed training protocol is outlined in Table 2.

**Table 2:** Experimental intervention protocol.

Group	Load	Sets	Repetitions	Load Increase Strategy
2/0/X/0	Load: 70% 1RM	First 4 weeks 3 sets, last 4 weeks 4 sets	Repetitions until failure to maintain tempo	Two-for-Two Rule
4/0/X/0	Load: 70% 1RM	First 4 weeks 3 sets, last 4 weeks 4 sets	Repetitions until failure to maintain tempo	Two-for-Two Rule
V/V/X/V	Load: 70% 1RM	First 4 weeks 3 sets, last 4 weeks 4 sets	Task failure	Two-for-Two Rule

**Table 3:** Participant information.

Basic Information	2/0/X/0	4/0/X/0	V/V/X/V	P-value
Number of participants	10	10	10	——
Body Mass (kg)	74 ± 3.3	80.5 ± 6.57	73.9 ± 3.84	——
Height (cm)	179.6 ± 3.27	181.7 ± 2.58	179.6 ± 3.0	——
Squat 1RM (pre)	131 ± 9.66	134.5 ± 13.6	132 ± 10.06	>0.05
30m Sprint (pre)	4.44 ± 0.18	4.41 ± 0.18	4.36 ± 0.23	>0.05
CMJ Height (pre)	45.75 ± 4.62	48.04 ± 5.93	43.88 ± 3.44	>0.05
SJ Height (pre)	46.10 ± 5.65	45.96 ± 5.65	45.92 ± 5.47	>0.05
Squat 1RM (post)	143.5 ± 7.47**	143.0 ± 12.74**	140.5 ± 8.64**	
30m Sprint (post)	4.32 ± 0.13**	4.53 ± 0.20*	4.39 ± 0.15*	
CMJ Height (post)	51 ± 5.06**	43.32 ± 4.42**	44.71 ± 1.78**	
SJ Height (post)	51.4 ± 4.21**	41.45 ± 4.39**	47.06 ± 3.06	
Repetitions	531.4 ± 63	416.8 ± 12.7	— ——	
TUT-E	1062.8 ± 126	1667.2 ± 50.8	——	

TUT-E:The duration of the eccentric contraction phase of muscles.

*:Significant within-group difference (P < 0.05).

**:Highly significant within-group difference (P < 0.01).

### Experimental control

To minimize interference from other factors in the experiment, the experimental site was consistently located in the Physical Fitness Monitoring Laboratory at the Rizhao Campus of Shandong Sport University. Experiments were scheduled for Tuesdays and Fridays from 2 PM to 4 PM. Participants were instructed to avoid alcohol and stimulant beverages, such as coffee, for 24 hours prior to the experiments. The same equipment was used for both pre-tests and post-tests to prevent errors due to equipment variability. To reduce confounding effects from other exercise stimuli, all participants were advised to refrain from lower body training other than the intervention throughout the study period.

## Statistical analysis

All data results in this study were expressed as mean ± standard deviation. Data were summarized using Excel 2021, and statistical processing of experimental results was performed using SPSS 26.0Before statistical analysis, the Shapiro-Wilks normality test was first conducted on the data of each group. Subsequently, a two-way analysis of variance (Two-Way ANOVA) was used to test the statistical significance of intergroup comparisons under different intervention methods. If the sphericity assumption test showed that P < 0.05 (indicating failure to meet the sphericity assumption) or P > 0.05 (indicating satisfaction of the sphericity assumption), a univariate test result was used. If the sphericity assumption was not satisfied, the Greenhouse-Geisser or Huynh-Feldt method was used for correction. The Bonferonni method was employed for post-hoc comparisons, with a significance level set at α < 0.05 for significant differences. Cohen’s d value was used to standardize the effect size (ES), where ES between 0.2–0.49 was defined as a small effect, 0.5–0.79 as a medium effect, and ≥0.8 as a large effect [32].

## Results

The Shapiro-Wilk test indicated that all pre-test indicators conformed to a normal distribution (P > 0.05). Participant information is shown in Table 3.

### Maximal strength

The results of two-way repeated measures ANOVA showed the following patterns in the change characteristics of maximum squat strength: The main effect of time was highly significant (F = 167.54, P < 0.001, η²p = 0.86), indicating that the strength level of all subjects significantly improved after 8 weeks of intervention. The main effect of group was not significant (F = 0.15, P = 0.86, η²p = 0.001). The time × group interaction was not significant (F = 3.08, P = 0.06, η²p = 0.18); however, this p-value approached the conventional significance threshold, and corresponding effect sizes indicated potentially meaningful trends, suggesting no between-group differences in the long-term effects of different movement tempos on maximum squat strength improvement. Post-hoc pairwise comparisons showed no significant differences between Group 2/0/X/0 and Group 4/0/X/0 (P = 0.911), Group 2/0/X/0 and the Group V/V/X/V (P = 0.503), or Group 4/0/X/0 and the Group V/V/X/V (P = 0.576). Within-group time effect analysis showed highly significant improvements before and after intervention in Group 2/0/X/0 (P < 0.001), Group 4/0/X/0 (P < 0.001), and the Group V/V/X/V (P < 0.001). In terms of within-group pre-to-post effect size assessment, the results were as follows: Group 2/0/X/0 (ES = 1.45)> Group V/V/X/V (ES = 0.85)> Group 4/0/X/0 (ES = 0.64).

### 30-meter sprint

A two-way repeated-measures analysis of variance (ANOVA) revealed a significant time × group interaction for 30-meter sprint time (F = 7.33, P = 0.003, η²p = 0.35), indicating that the improvement effects of different movement tempos on 30-meter sprint performance exhibited between-group differentiation over time. Neither the main effect of time (F = 0.02, P = 0.87, η²p = 0.001) nor the main effect of group (F = 0.98, P = 0.39, η²p = 0.068) reached statistical significance. Post-hoc pairwise comparisons between groups showed that Group 2/0/X/0 was significantly superior to Group 4/0/X/0 (P = 0.007), while there was no significant difference between Group 2/0/X/0 and the Group 4/0/X/0. The analysis of the within-group time effect demonstrated that Group 2/0/X/0 showed significant improvement (P = 0.009), while Group 4/0/X/0 showed a significant decline (P = 0.017) in 30-meter sprint performance after the intervention, whereas no significant change was observed in the Control Group (P = 0.59). Specifically, the ranking of intervention effects based on effect size was as follows: Group 2/0/X/0 (ES = −0.76)> Group V/V/X/V (ES = −0.1)> Group 4/0/X/0 (ES = 0.63).

### CMJ

Similar to the 30-meter sprint, the two-way repeated measures ANOVA results showed a highly significant time × group interaction for CMJ height (F = 36.38, P < 0.001, η²p = 0.73), indicating that the improvement effects of different movement tempos on CMJ height demonstrated between-group differences over time. Neither the main effect of time (F = 0.84, P = 0.37, η²p = 0.03) nor the main effect of group (F = 2.38, P = 0.11, η²p = 0.15) reached statistical significance. Post-hoc pairwise comparisons revealed highly significant differences between Group 2/0/X/0 and Group 4/0/X/0 (P < 0.001), highly significant differences between Group 2/0/X/0 and the Group V/V/X/V(P = 0.002), and no significant difference between Group 4/0/X/0 and the Group V/V/X/V (P = 0.44), suggesting that Group 2/0/X/0 improved CMJ height, while the control group had no significant effect on CMJ height. Within-group time effect analysis showed that the time factor had a highly significant effect on CMJ height in both Group 2/0/X/0 and Group 4/0/X/0 (P < 0.001), but it should be noted that Group 4/0/X/0 showed a significant decrease after 8 weeks of intervention (ES = −0.9). No significant difference was observed in the control group (P = 0.32). In terms of effect size, the results were as follows: Group 2/0/X/0 (ES = 1.07)> Group V/V/X/V (ES = 0.3)> Group 4/0/X/0 (ES = −0.9).

### SJ

The two-way repeated measures ANOVA results showed a time × group interaction effect for SJ height (F = 27.93, P < 0.001, η²p = 0.67), indicating that the effects of different movement tempo trainings on SJ height demonstrated between-group differences over time. Neither the time effect (F = 1.65 P = 0.2, η²p = 0.058) nor the main effect of group (F = 2.80, P = 0.08, η²p = 0.17) reached statistical significance. Post-hoc pairwise comparisons revealed highly significant differences between Group 2/0/X/0 and Group 4/0/X/0 (P = 0.002), as well as between Group 4/0/X/0 and the control group (P = 0.005). Within-group time effect analysis showed that the time factor had a highly significant effect on both Group 2/0/X/0 and Group 4/0/X/0 (P < 0.001), while there was no significant difference in the control group (P = 0.03). In terms of effect size, the results were as follows: Group 2/0/X/0 (ES = 1.09)> Group V/V/X/V (ES = 0.34)> Group 4/0/X/0 (ES = −0.83).

## Discussion

The tempo control of different muscle contraction phases directly affects Time Under Tension (TUT). Prolonged TUT significantly increases metabolic stress, which has been confirmed as a key factor influencing muscle strength development [17]. Muscle contraction phases are typically divided into eccentric, isometric, and concentric contractions. Previous studies have mostly focused on the effects of TUT in each phase on strength, with eccentric contraction tempo receiving particular attention—owing to its higher efficiency in promoting protein synthesis responses and positively regulating anabolic signaling pathways and gene expression [23]. However, fewer studies have explored the combined effects of eccentric tempo and explosive concentric contraction. Given that explosive contraction intent is critical for driving neural adaptation and strength gains [19], this study compared training modes of “different eccentric tempos + explosive concentric contraction” versus “ self-selected eccentric, isometric, and inter-repetition tempos, with fixed explosive concentric tempo “to investigate the effects of different movement tempos on lower limb muscle strength and power. The results showed: 1) Different eccentric tempos combined with explosive concentric contraction showed no statistical difference in maximum strength compared with self-selected tempo, but the former had higher ES values; 2) Significant between-group differences were observed in power indices: Group 2/0/X/0 showed significantly better performance in CMJ height, SJ height, and 30-meter sprint than other groups; the control group showed significant improvements in CMJ and SJ heights, with no change in 30-meter sprint; Group 4/0/X/0 showed significant declines in power indices. This suggests that the explosive intent during the concentric phase cannot fully offset the negative impact of prolonged eccentric tempo on power.

### Maximum squat strength

Maximum strength is a key determinant of power performance—athletes with higher strength levels typically exhibit better power output capabilities. For sports requiring explosive power, developing maximum strength serves as the core component of physical training programs [30]. This study found that maximum squat strength significantly improved in all groups (P < 0.01), but no statistical differences were observed between groups. This finding aligns with Davies et al. [33], who suggested that maximum strength gains from fast or slow tempos show no statistical differences. However, effect size analysis revealed Group 2/0/X/0 (ES = 1.45)> Group V/V/X/V (ES = 0.85)> Group 4/0/X/0 (ES = 0.64), indicating that rapid eccentric contraction combined with explosive concentric intent maximizes strength gains. Notably, the control group showed a larger effect size than Group 4/0/X/0—an unexpected result. It should be noted that although the control group used self-selected contraction tempo, their eccentric contraction time was significantly shorter than Group 4/0/X/0. This finding contrasts with Mike et al.[19], whose study using slow eccentric tempo (6/1/2) achieved the highest ES (0.88), with ES increasing as eccentric time prolonged (6/1/2 > 4/1/2 > 2/1/2). This suggests that longer eccentric tempos correlate with more significant maximum strength gains, consistent with most previous studies: while fast and slow eccentric trainings show no significant between-group differences in maximum strength gains, slow eccentric tempos combined with fast concentric tempos yield better results [12,22,34]. This finding supports the initial hypothesis: longer eccentric tempos increase muscle tension, triggering muscle fiber structural remodeling—a key mechanism for muscle hypertrophy. Given the significant positive correlation between muscle cross-sectional area (CSA) and maximum strength, such structural adaptations ultimately promote strength gains [35]. However, this study found that Group 2/0/X/0 had a significantly higher ES than Group 4/0/X/0 and the Group V/V/X/V, differing from initial expectations. We specifically adopted unified explosive concentric execution across all experimental groups in this research design. Such arrangement was made to eliminate confounding effects brought by varied concentric movement patterns, so that we could independently explore the adaptive effects of different eccentric contraction durations when matched with consistent fastest-speed concentric contraction on maximal strength development.

Against this unified experimental premise, the superior strength adaptation efficiency of the 2/0/X/0 group may be largely attributed to its moderate eccentric duration. This tempo mode can facilitate sufficient pre-stretch stimulation and elastic energy accumulation in skeletal muscle, and may avoid excessive time-under-tension load and subsequent potential neuromuscular inhibition induced by the ultra-long eccentric phase of the 4/0/X/0 protocol. This kind of eccentric duration matching pattern is more likely to fit the physiological adaptation rules of lower limb muscles, which may partly account for its relatively better strength improvement effect. Furthermore, properly controlled eccentric loading duration may optimize motor unit recruitment mode and intramuscular coordination state, which could further form differentiated strength adaptation outcomes among different tempo schemes.

Additionally, subjects’ training experience is a critical factor. Most relevant existing literature selects well-resistance-trained participants as research objects, while the subjects recruited in this study are mainly professional sports college students with relatively distinct training foundations and adaptive characteristics [12,19,22,23]. Such population differences may also partly explain the inconsistent research conclusions compared with previous studies, highlighting the need for future research to carry out targeted exploration aiming at specific athlete groups.

### Lower limb power

#### Vertical jump height.

In physical performance-dominant sports, an athlete’s ability to efficiently utilize the Stretch-Shortening Cycle (SSC) is critical for enhancing running economy, among other skills. In training practice, CMJ and SJ tests serve as effective means to assess an athlete’s SSC capability [36]. Specifically, the SSC consists of three phases: 1) Eccentric contraction, where the agonist muscles bear preload, energy is stored in the Series Elastic Component (SEC),and muscle spindle receptors are stimulated; 2) Transition phase (from eccentric to concentric contraction), where signals travel via Ia afferent nerve synapses to α-motor neurons, which then transmit signals to the agonist muscles; 3) Concentric phase, where energy stored in the SEC during the eccentric phase is released to enhance subsequent movements [30].

This study found that CMJ and SJ heights significantly increased in Group 2/0/X/0 and the Group V/V/X/V, while Group 4/0/X/0 showed a significant decrease. This contrasts with the strength gains in Group 4/0/X/0—typically, improved maximum strength predicts better vertical jump performance [37]. This phenomenon may stem from two factors: 1) Prolonged TUT increases Type I muscle fiber recruitment, reducing SSC utilization; 2) Excessively long eccentric contraction times fail to sufficiently stimulate muscle spindles, inhibiting concentric force production [38]. The divergent adaptations—improved maximal strength yet impaired explosive performance in the 4/0/X/0 group—may be explained by four interrelated mechanisms. First, prolonged eccentric duration prolongs time under tension and reduces the efficiency of the stretch-shortening cycle (SSC), weakening muscle spindle stimulation and rapid elastic recoil. Second, as direct measurements of motor unit activation and rate of force development (RFD) were not collected in this study, we present this as a hypothesized physiological mechanism: excessively slow eccentric loading may preferentially recruit type I muscle fibers and reduces fast-twitch motor unit activation, which could in turn lower RFD and contribute to diminished explosive output. Third, prolonged eccentric contraction may alter trunk and lower-extremity mechanics during jumping and sprinting, reducing measured vertical displacement and linear speed without necessarily reducing intrinsic muscle force. Fourth, prolonged mechanical tension may induce partial neuromuscular inhibition that selectively impairs rapid concentric expression, even while maximal voluntary strength is improved. Together, these mechanisms explain why strength can increase while explosive performance declines following slow-tempo eccentric training [21,38].

Notably, the observed divergence between increased maximal strength and reduced jump height in the 4/0/X/0 group aligns with the key training principle proposed by Michael Boyle: “train slow, move slow” [39]. Following 8 weeks of chronic prolonged eccentric tempo training, consistent exposure to slow, controlled muscle actions may reduce the efficiency of rapid eccentric-to-concentric switching, thereby impairing explosive stretch-shortening cycle (SSC) function. While prolonged eccentric training is recognized to enhance eccentric muscle strength and loading tolerance—qualities considered important for long-term explosive development in periodized training models—the present findings indicate that exclusive reliance on slow eccentric training can compromise rapid power output despite improvements in maximal strength. The reduction in CMJ and SJ height may therefore represent a predictable, mechanism-based trade-off: enhanced maximal strength and eccentric capacity from prolonged time under tension, at the cost of rapid SSC efficiency. As noted earlier, these interpretations should be tempered by the limitations of displacement-only measurement and the heterogeneous movement backgrounds of the sample, which may further influence observed jump mechanics.

Similar to our results, Segers et al. [21] investigated different movement tempos in elite soccer players during the competitive season and found that the slow eccentric tempo group had lower vertical jump heights than the fast tempo group. Mike et al. [19] also reported that slow eccentric tempo training reduced peak takeoff velocity. In contrast, Pierpaolo et al. [38] found positive effects of slow resistance training on CMJ height, hypothesizing that slow training induces muscle oxygen deficit faster, promoting early muscle fiber recruitment—a mechanism similar to blood flow restriction training, though unconfirmed. Additionally, Pierpaolo used seated leg extensions, a single-joint movement differing significantly in neural adaptation from the multi-joint squat used in this study. Considering exercise-specific training effects, the present findings do not fully support their conclusion.. Although prolonged eccentric tempos may hinder vertical jump height gains, some studies suggest such training enhances eccentric energy storage for greater concentric force output, warranting further investigation [1].

Notably, pre-test CMJ and SJ heights showed minimal differences among subjects—an unexpected result. SJ relies primarily on concentric muscle contraction without pre-stretch, while CMJ generates greater power by leveraging muscle elasticity through rapid eccentric-stretch followed by concentric contraction. Thus, CMJ performance should exceed SJ. In addition to the tempo intervention effects, we also found significant inter-group differences between the 4/0/X/0 group and the self-selected tempo control group in SJ performance. This further verified that long-duration eccentric contraction intervention (4/0/X/0) failed to induce superior jump ability adaptation; conversely, participants under self-regulated training mode achieved better jump performance improvements. Such findings indicate that excessively slow eccentric tempo may restrict the adaptive benefits of resistance training on lower-limb explosive force, and autonomous training rhythm is more conducive to maintaining and promoting stretch-shortening cycle function.

However, it should be acknowledged that the present study only measured vertical jump height using a linear position transducer (VBT Pro), which solely quantifies vertical displacement without capturing full-body kinematics or movement technique changes. As highlighted in a recent scoping review on artificial intelligence in sports biomechanics [40], displacement-based devices alone cannot differentiate between genuine physiological changes in explosive power production and alterations in movement mechanics or jumping technique. Comprehensive motion analysis systems, such as marker-based or markerless motion capture, are necessary to obtain detailed kinematic data and clarify whether the observed declines in jump height in the 4/0/X/0 group arose from impaired SSC function, modified movement patterns, or a combination of both. Future studies combining motion analysis with linear position transducer testing would enable more robust differentiation between physiological adaptations and technique-related changes, as emphasized in contemporary biomechanical assessment frameworks [40].

#### 30-meter sprint speed.

Short-distance sprinting serves as a common indicator for evaluating lower limb power [5,38,41].Similar to vertical jump, maximum strength shows a positive correlation with sprint performance [24]. Studies indicate that in young athletes or low-strength populations, maximum strength training alone can improve power output, but as strength levels increase, continued maximum strength development may lead to diminishing returns in power gains [42]. In this study, all groups showed significant maximum strength improvements, theoretically suggesting potential sprint performance enhancements for all three groups. However, the results were similar to those of the vertical jump metrics: only Group 2/0/X/0 showed significant improvement, while both Group 4/0/X/0 and the Group V/V/X/V exhibited no significant changes. Given no pre-test differences in maximum strength among groups, this cannot be attributed to Group 4/0/X/0 or the Group V/V/X/V reaching a diminishing returns threshold due to higher strength levels. Although rate of force development (RFD) was not tested, we speculate that slow eccentric training prolonged RFD during sprinting, negatively impacting speed. This is supported by Häkkinen et al. [43] and Komi et al. [44], who found untrained individuals and heavy resistance trainers achieved similar maximum RFD within 200ms, while explosive-trained individuals showed significantly faster RFD. Although none of the three groups performed explosive exercises, Group 2/0/X/0’s movement tempo was relatively the shortest, potentially activating high-threshold motor units and enhancing concentric RFD to a greater extent [38].

Segers et al. [21] compared 1/0/1/0 vs. 4/0/1/0 tempo resistance training in elite soccer players and found no significant between-group differences in 20-meter sprint performance. This may be due to the short 5-week intervention period being insufficient to induce significant improvements in top-level athletes. Similarly, Brien et al. [41] observed no sprint performance gains after 4 weeks of slow eccentric training (4/0/2/0) in female basketball players. Pierpaolo et al. [38] presented an interesting contrast: the slower 3/0/3/0 tempo yielded significantly better 30-meter sprint improvements than 1/0/1/0. Notably, their study was conducted during the competitive season, with subjects engaging in 5 weekly training sessions and one match, which may have confounded results.

On another note, Cook et al. [45] found no 40-meter sprint changes after 3 weeks of pure eccentric(3s) resistance training in youth rugby players. However, combining slow eccentric resistance training with overspeed training significantly improved sprint speed [43]. This may occur because slow eccentric training enhances muscle force absorption, while overspeed training optimizes SSC capability, synergistically improving sprint performance. This finding suggests a new research direction: investigating the combined effects of slow eccentric resistance training and overspeed training on short-distance sprint performance.

## Conclusion

At 70% 1RM load, free-weight squat training with a 2/0/X/0 tempo significantly improves lower limb maximum strength, CMJ height, SJ height, and 30-meter sprint performance in university students majoring in sports training compared with 4/0/X/0 and V/V/X/V tempos. In addition, the control group showed superior SJ performance compared with the 4/0/X/0 group, further indicating that ultra-slow eccentric loading is not suitable for explosive strength training intervention. Based on this, it is recommended that strength and conditioning coaches prioritize squat training with a 2/0/X/0 tempo when designing lower limb strength programs to maximize the benefits for lower limb muscle strength and power development.

## Limitations

The results of this experiment are primarily applicable to individuals with a foundation in resistance training, and the effects on other training-level populations require further practical validation. Additionally, the following limitations should be noted:

(1) Lack of plyometric training control group: For individuals with weak eccentric capabilities, combining slow eccentric training with plyometric training may be more effective for power development, a hypothesis that warrants further verification.
(2) Practical limitations in movement tempo control: Although isometric contraction, concentric contraction, and inter-movement tempo were designed as 0 or X seconds in the experiment, actual movement transitions inevitably occupy time, introducing errors in metronome tempo design.
(3) Technical issues under specific tempo and load: Isolated instances of barbell slippage were observed in a small number of stronger participants within the 2/0/X/0 group during the eccentric phase of squats. Such rare occurrences may have temporarily compromised trunk rigidity in those individuals, thereby potentially influencing force transmission efficiency during those specific repetitions. Given the infrequent nature of this issue and strict supervision of movement technique, it is not believed to have systematically confounded the overall group-level outcomes.
(4) All participants were male: This study included only male subjects, limiting the generalizability of results to female populations. Given gender-specific sensitivities to resistance training, future research should expand gender diversity to clarify the effects of movement tempos on different genders and provide more universal findings.
(5) Baseline imbalance in vertical jump performance: The narrow baseline difference between CMJ and SJ heights represented a significant confounding factor in this study. This pattern may be partly attributed to the heterogeneous athletic backgrounds of participants (e.g., throwing, 400 m running, rowing, boxing, handball) and their self-reported prior training emphasis on concentric contraction, rather than eccentric or rapid eccentric–concentric transition abilities. This limitation may reduce the internal validity of jump performance comparisons and could constrain the generalizability of the present findings.
(6) Vertical jump testing constraint: The PVC pole used to fix the linear position transducer restricted arm swing in CMJ and SJ tests, which altered standard jump biomechanics and may limit the generalizability of the explosive performance findings.

## Supporting information

S1 File Raw Data. All original experimental measurement data obtained from participants in this study.
(XLSX)
